# Acetylome analyses provide novel insights into the effects of chronic intermittent hypoxia on hippocampus-dependent cognitive impairment

**DOI:** 10.3389/fnmol.2024.1324458

**Published:** 2024-02-22

**Authors:** Fan Liu, Weiheng Yan, Chen Chen, Yubing Zeng, Yaru Kong, Xuejia He, Pei Pei, Shan Wang, Ting Zhang

**Affiliations:** ^1^Children’s Hospital Capital Institute of Pediatrics, Chinese Academy of Medical Sciences and Peking Union Medical College, Beijing, China; ^2^Beijing Municipal Key Laboratory of Child Development and Nutriomics, Capital Institute of Pediatrics, Beijing, China; ^3^Graduate School of Peking Union Medical College, Beijing, China; ^4^Beijing Municipal Key Laboratory of Child Development and Nutriomics, Capital Institute of Pediatrics-Peking University Teaching Hospital, Beijing, China

**Keywords:** cognition, lysine acetylation, CIH, hippocampus, mitochondria

## Abstract

**Introduction:**

Chronic intermittent hypoxia (CIH) can negatively affect hippocampal function through various molecular mechanisms. Protein acetylation, a frequently occurring modification, plays crucial roles in synaptic plasticity and cognitive processes. However, the global protein acetylation induced by CIH in the hippocampus and its specific effects on hippocampal function and behavior remain poorly understood.

**Methods:**

To address this gap, we conducted a study using liquid chromatography-tandem mass spectrometry to analyze the lysine acetylome and proteome of the hippocampus in healthy adult mice exposed to intermittent hypoxia for 4 weeks (as a CIH model) compared to normoxic mice (as a control).

**Results:**

We identified and quantified a total of 2,184 lysine acetylation sites in 1,007 proteins. Analysis of these acetylated proteins revealed disturbances primarily in oxidative phosphorylation, the tricarboxylic acid (TCA) cycle, and glycolysis, all of which are localized exclusively to mitochondria. Additionally, we observed significant changes in the abundance of 21 proteins, some of which are known to be associated with cognitive impairments.

**Discussion:**

This study helps to elucidate the molecular mechanisms underlying CIH-induced changes in protein acetylation in the hippocampus. By providing valuable insights into the pathophysiological processes associated with CIH and their impacts on hippocampal function, our findings contribute to a better understanding of the consequences of CIH-induced changes in protein acetylation in the hippocampus and the potential role of CIH in cognitive impairment.

## 1 Introduction

Obstructive sleep apnoea (OSA) is a typical sleep disorder characterized by recurrent episodes of pharyngeal collapse during sleep. Moreover, OSA causes repetitive fluctuations in blood oxygen saturation and leads to chronic intermittent hypoxia (CIH). Over the past two decades, the prevalence of OSA has doubled (Bannow et al., 2022). Cognitive and behavioral scales have indicated OSA-induced abnormalities in learning, memory, and cognition (Hunter et al., 2016; Labarca et al., 2020; Osorio et al., 2022). In addition, brain MRI revealed OSA-associated reductions in the frontal cortex, anterior cingulate cortex, and hippocampus (Canessa et al., 2011; Zhao et al., 2016; Philby et al., 2017; Koo et al., 2020). Research has indicated that patients with OSA exhibit localized reductions in the volume of gray matter within the hippocampus (Canessa et al., 2011) and significant decreases in hippocampal neuronal functional connectivity (Zhou et al., 2020). The brain constitutes 20% of basal oxygen utilization, rendering it exceptionally susceptible to hypoxic conditions (Burtscher et al., 2021). OSA imposes brain risks through CIH and impairs cognitive performance. Although earlier studies have shown that OSA impacts adult hippocampal neurogenesis and cognitive processes (Anacker and Hen, 2017), the exact mechanism through which CIH affects hippocampal function, particularly learning and memory, remains unclear.

As neuroinflammation can influence cognitive functions, it may constitute a significant mechanism underlying the cognitive deficits induced by CIH. Cao et al. (2020, 2021) ascertained that aberrant autophagic activity within hippocampal neurons is associated with impaired cognitive function. Upon suppression of excessive autophagy, apoptosis of hippocampal neurons is ameliorated. Substantial astrogliosis within the cortical and hippocampal regions of rats subjected to IH has been documented (Aviles-Reyes et al., 2010). Microglia play key physiological roles, including synapse monitoring, debris clearance, and synaptic pruning. They impact cognition by modulating learning and memory via neuronal activity and synaptic plasticity (Ben Achour and Pascual, 2010; Yang et al., 2010). CIH triggers neurocognitive impairments in the hippocampus by enhancing neuroinflammation, neuroapoptosis, and oxidative stress (Zhou et al., 2016).

Posttranslational modifications (PTMs) are critical for regulating various cellular processes, including protein-protein interactions, enzyme activity, and gene expression. The eukaryotic proteome consists of hundreds of distinct PTMs. However, only a few proteins, such as those involved in phosphorylation, glycosylation, methylation, ubiquitylation, and acetylation, have been extensively investigated (Narita et al., 2019). Among the many types of PTMs that occur in proteins, lysine acetylation plays critical roles in regulating memory and the balance between neuroprotection and neurodegeneration (Schueller et al., 2020; Qian et al., 2022). Lysine acetylation is a reversible PTM that affects protein function through various mechanisms, including altering charge, structure, stability, and interactions with other molecules. Moreover, it has been shown to regulate numerous cellular processes, including gene expression, chromatin remodeling, metabolism, and mitochondrial function (Xiao et al., 2020). Recently, considerable focus has been directed toward the functions of hypoxia-induced PTMs in various pathological conditions. However, less is known about possible CIH-induced PTMs. Histone acetylation is involved in memory and long-term synaptic plasticity (Mews et al., 2017; Campbell and Wood, 2019). Spatial memory relies on changes in gene expression in the hippocampus, which are partly regulated by histone acetylation (Mews et al., 2017). Specifically, dysregulation of H3K9 acetylation has been associated with impaired establishment of epigenetic memory at genes involved in striatal plasticity (Alcalá-Vida et al., 2022). Additionally, inhibiting the histone deacetylase (HDAC) family with sodium butyrate (NaB) administration attenuated neurodegeneration and memory loss in hypobaric hypoxia-exposed rats (Kumar et al., 2021). Recently, non-histone acetylation has gained increased amounts of attention. SIRT1 ameliorated CIH-induced cognitive behaviour in mice by reducing NF-κB acetylation in the hippocampus (Fan et al., 2018). By deacetylating the RelA/p65 subunit of NF-κB at lysine 310, SIRT1 can suppress its transcriptional activity and reduce the expression of proinflammatory genes (Yeung et al., 2004). SIRT1 activation significantly promoted potent neuroprotection (Chen et al., 2005). SIRT1 deficiency in microglia leads to the upregulation of IL-1β, resulting in cognitive decline (Cho et al., 2015). Although acetylation is known to be associated with hippocampal cognitive function, the relationships between acetylation, especially non-histone acetylation, and hippocampal function in CIH patients have not been elucidated.

In this study, we employed liquid chromatography–tandem mass spectrometry (LC-MS/MS) to investigate whether CIH alters the hippocampal acetylation landscape. Quantitative analysis of the acetylome revealed the involvement of acetylated proteins in oxidative phosphorylation and the TCA cycle, primarily in mitochondria, linking CIH to cognitive function.

## 2 Materials and methods

### 2.1 Animals

This study utilized male C57BL/6J mice that were obtained from Sibeifu Biotechnology Co., Ltd. The mice were 6 weeks old and weighed between 20–22 g at the beginning of the experiment. To ensure their health and wellbeing, all mice used in the study were free from specific pathogens. Throughout the experimental period, the mice were housed in a controlled environment with a 12/12-h light/dark cycle. They were provided with *ad libitum* access to food and water. To maintain stable conditions, the temperature and humidity were strictly controlled. After being habituated to their new environment for 1 week, the mice were randomly assigned to either the CIH or control (CON) group. All procedures were carried out during the mice’s inactive period and their body weight was monitored weekly. The Capital Institute of Pediatrics’ Ethics Committee on Animal Care and Use approved the study on November 9, 2021 (approval No. DWLL2021016).

### 2.2 Establishment of chronic intermittent hypoxia model and supplementation of sodium butyrate

We followed an established CIH modeling method for gas control (Du et al., 2020; Hernández-Soto et al., 2021). Mice were kept in custom standard cages (Zhongshi Technology Co, Ltd). A gas control system managed room airflow (N_2_ and O_2_). Programs and flow regulators allowed manipulation of inspired O_2_ fraction from 20.9 to 5.0% over 2 min, followed by rapid reoxygenation to normal air levels via a 100% O_2_ burst in the next minute. Regarding the duration and timing of the CIH protocol, we have made improvements based on a previous CIH modeling method (Du et al., 2020; Hernández-Soto et al., 2021). Intermittent hypoxia events occurred cyclically for 8 h each day, from 9:00 am to 5:00 pm, during the light phase and lasted for 28 days. At other times, CIH animals were in a normoxic environment. Control animals were in a normoxic chamber for 28 days. The animals’ weight and survival were monitored during this protocol. One day after chronic intermittent hypoxia modeling was completed, mice from both the CON and CIH groups was euthanized simultaneously to collect tissues for further experiments, including histological staining and omics sequencing analyses. The remaining mice were kept for behavioral experiments, including the Novel Object Recognition Test (NORT) and Y-maze test, with at least 1 day of rest between the two tests.

After the 28-day CIH protocol, half of the mice in the CIH group were randomly selected to receive treatment with NaB, forming the CIH+NaB group. The mice in this group were administered intraperitoneal injections of NaB (300 mg/kg, Sigma-303410) at a dosage of 100 μl once daily for a consecutive period of 14 days. On the 15th day, NORT tests or other types of analysis were performed.

### 2.3 Measures of metabolic parameters

For the locomotion assay, the animals were placed in an open box (50 × 50 × 35 cm, Beijing Zhongshi Dichuang Technology Development Co., Ltd) and allowed to freely move for 5 min after completing the 28-day CIH protocol (*n* = 12 mice/group). The distance traveled by each animal during this 5-min period was recorded using a camera. Food and water intake measurements were taken for a 16-h period immediately following the 28-day CIH exposure (Ciriello et al., 2021). For all analyses, the experimental unit used was mice, except for food/water consumption where the cage (with 4 mice per cage) served as the experimental unit. This was due to the inability to measure individual food and water consumption in standard individually ventilated cages.

### 2.4 Animal behavioral assessment

The cognition of the mice was evaluated using the NORT and open Y-maze after the CIH process. The mice were acclimated to the testing room and the apparatuses were cleaned before each test. A night vision camera recorded their activities and a blinded investigator carried out all assessments and data analyses. 12 mice in each group were chosen for the experiments. Inactive mice were excluded.

#### 2.4.1 Novel object recognition test

Novel object recognition test (NORT) was performed in an open box (50 × 50 × 35 cm, Beijing Zhongshi Dichuang Technology Development Co., Ltd). During the adaptation stage, two objects with identical shape and material were positioned in the symmetrical area. In the open field test, each mouse was placed in the center of the open box and allowed to explore the two objects for 5 min while their behavior was recorded. Taking out the mice and detecting the recognition period after an interval of 1 h. In the recognition stage, we replaced one object (Green) with a new different object (Red), then repeat the procedure. The mice were again put into the open field to explore freely. The camera recorded the time exploring a new object (TN) and time exploring a familiar object (TF) of object A within 5 min, and the software (Beijing Zhongshi Dichuang Technology Development Co., Ltd) was used to track the mouse’s trajectory. New thing identification index = [(TN-TF)/(TN+TF)] × 100%, the higher the index, the better the memory of mice.

#### 2.4.2 Y-maze test

We utilized an experimental setup called the Y-maze, which consists of three identical arms measuring 300 × 200 × 60 mm each. The mice were placed in an arm and their movements were recorded while they explored for 8 min. To determine spontaneous alternation behavior, we counted the number of times a mouse consecutively entered all three arms of the maze. A higher percentage of spontaneous alternations indicates better spatial working memory performance. We calculated the percentage of spontaneous alternation using the formula [number of spontaneous alternations / (total arm entries–2)] × 100.

### 2.5 Hematoxylin–Eosin (HE) and Nissl staining

Six Mice from each group without undergoing behavior test were anesthetized with chloral hydrate and then sacrificed. To evaluate histological damage, mice were perfused with saline and paraformaldehyde. Their brains were removed, fixed in paraformaldehyde for 24 h, dehydrated in alcohol, and embedded in wax. The wax was trimmed and sectioned into 4 μm slices for staining with HE and Nissl. Then, the sections were dewaxed and dehydrated using xylene and ethanol solutions before being rinsed with tap water. For HE staining, they were stained with hematoxylin solution (Servicebio, G1003) and treated with differentiation and bluing solutions before being fixed with ethanol and stained with Eosin dye. The sections were then dehydrated and placed in xylene before being sealed with neutral gum. For Nissl Staining, they were stained with Nissl dye (Servicebio, G1036) and treated with a differentiation solution before being rinsed and sealed with neutral gum. The pathological changes in the hippocampus were observed under a light microscope. The Nissl-stained positive neurons in the hippocampal dentate gyrus (DG) region were counted under a light microscope. Besides, each section were visually counted in a blinded manner. The results show the different number of surviving neurons in CIH group compared to the CON group in same regions (Chu et al., 2019; Ke et al., 2020).

### 2.6 Multiplex immunofluorescence staining

Each group (*n* = 6 mice/group) without undergoing behavior test were anesthetized with chloral hydrate and then sacrificed. To prepare the tissue sections for immunohistochemistry, we treated them with a 0.3% hydrogen peroxide solution and then used microwave treatment to enhance antigen retrieval. We blocked the sections in 5% BSA before incubating them overnight at 4°C with the primary antibody GFAP (diluted to 1:100, Cell Signal Technology). The next day, we performed secondary antibody detection using HRP-conjugated anti-rabbit IgG (ZSGB-Bio, Beijing, China) at room temperature for 1 h. We utilized TSA reaction of AlexaFluor FITC-Conjugated TSA (1:50, Akoya) to visualize immunoreactivity, followed by microwave treatment for 15 min and cooling. Subsequently, we performed immunostaining with the primary antibodies Neun (diluted to 1:1000, Cell Signal Technology), Iba-1 (diluted to 1:200, Cell Signal Technology), and DCX (diluted to 1:200, Cell Signal Technology) successively on the same section. Corresponding secondary detections were performed with AlexaFluor CY3- and CY5-Conjugated TSA (1:50, Akoya). For image capture, a fluorescence microscope (Olympus BX43 microscope) was used under uniform exposure settings and conditions for all samples. Subsequently, the acquired images were processed using ImageJ software^1^ in a blind manner. To assess the staining intensity of Neun, GFAP, Iba-1, and DCX, we measured the integrated density (IntDen). Utilizing ImageJ software, positive staining was quantified in terms of pixels, and then IntDen (calculated as the area multiplied by the mean gray value) (Mela et al., 2022), was determined as an indirect indicator of protein level.

### 2.7 Cytokine and chemokine assays

In the central nervous system (CNS), glial cells could mediate the neuroinflammation by releasing potentially neurotoxic mediators including cytokines, chemokines. In order to analyze these inflammatory cytokines, we performed luminex liquid suspension chip assay by Wayen Biotechnologies (Shanghai, China), including interleukin (IL)-4, IL-6, IL-10, and tumor necrosis factor (TNF)-alpha. Briefly, we obtained hippocampus tissue samples from the CIH and CON groups (3 mice per group). We lysed and centrifuged the samples at 13,200 rpm for 15 min. After measuring the protein concentrations, we diluted 45 μg total protein in 50 μl solution to ensure equal protein quantity and equal buffer volume for each sample. After incubating the samples in 96-well plates with embedded microbeads for 1 h, we added detection antibodies (anti-mouse 31 cytokines, as instructed in the Luminex 200 kit manual) and incubated them for an additional 30 min. Subsequently, we introduced streptavidin-PE to each well and incubated the mixture at 850 rpm for 10 min. These samples were then incubated in 96-well plates embedded with microbeads for 1 h. Subsequently, detection antibodies (anti-mouse 31 cytokines, according to the manual of the Luminex 200 kit) were added and incubated for an additional 30 min. Finally, streptavidin-PE was added to each well and incubated at 850 rpm for 10 min. The values were measured using the Bio-Rad Luminex Bio-Plex 200 System.

### 2.8 Sample collection, protein extraction, and trypsin digestion

After the CIH procedure, the mice were euthanized, and their brains were extracted. Six mice were used for the CON group and another six mice for the CIH group. Due to the relatively small volume of the hippocampus, a strategy was employed to pool the hippocampi from two mice together as one replicate, and three replicates were conducted for each group. Specifically, the hippocampi were isolated and quickly frozen using liquid nitrogen to limit degradation. To avoid the potential influence of circadian rhythms, tissue collection was conducted simultaneously, ensuring that all samples were obtained at the same time. The samples were then stored at −80°C until processed further. Subsequently, the cellular powder was treated with lysis buffer and a protease inhibitor, followed by sonication utilizing a high-intensity ultrasonic processor (Scientz). After removing debris through centrifugation at 12,000 *g* for 10 min at 4°C, the supernatant containing the protein solution was obtained and quantified for its protein concentration using BCA kit. The protein solution underwent treatment with 5 mM dithiothreitol at 56°C, followed by alkylation with 11 mM iodoacetamide at 25°C in the absence of light. To minimize the urea concentration to less than 2 M, 100 mM TEAB was added to dilute the protein sample. For the initial overnight digestion, trypsin was employed at a 1:50 ratio of trypsin-to-protein mass to initiate digestion, and peptides were desalted by C18 solid-phase extraction column. The method for extracting and breaking down proteins was identical for both the proteome and acetylome.

### 2.9 Acetylated peptide enrichment and LC-MS/MS analysis

To enrich acetylated peptides in the acetylome, we followed a similar protocol as described above for protein extraction and trypsin digestion. However, we included additional inhibitors (3 μM trichostatin and 50 mM nicotinamide) in the lysis buffer, and each sample utilized 2.5 mg of protein for trypsin digestion. Subsequently, we dissolved the peptides in NETN buffer (1 mM EDTA, 100 mM NaCl, 0.5% NP-40,50 mM Tris–HCl, pH 8.0) and incubated them overnight with anti-acetyllysine antibody-conjugated agarose beads. Once the beads were washed and the bound peptides were eluted using trifluoroacetic acid, we combined the eluted peptides, vacuum-dried them, desalted them, and prepared them for analysis. More specific formulations for the inhibitors and NETN buffer can be found elsewhere (Qian et al., 2022). LC-MS/MS analysis was conducted at PTM Biolab in Hangzhou, China. We dissolved the desalted peptides in solvent A, which contained 0.1% formic acid in 2% acetonitrile. Using a reversed-phase analytical column and a gradient of solvent B (0.1% formic acid in 100% acetonitrile), we performed proteome analysis with a gradient ranging from 6 to 24% over 70 min, followed by an increase from 24 to 35% over 12 min, and finally reaching 80% over 4 min, holding at 80% for an additional 240 s, while maintaining a constant flow rate of 450 nL/min. For acetylome analysis, we used a gradient starting at 6% and increasing to 24% over 40 min. The TimsTOF Pro mass spectrometer from Bruker Daltonics was used to analyze the peptides.

To analyze the raw MS/MS data obtained from our proteomic analysis, we utilized the MaxQuant computational proteomics platform (version 1.6.15.0^2^). The MaxQuant platform implements the MaxLFQ algorithm, and the specific algorithmic rules have been previously reported (Cox et al., 2014). We compared the data against the Mus_musculus_10090_SP_ 20230103.fasta database (Uniprot, 17,132 entries, acquired on 2023.1.3), which includes common contaminants and a reverse decoy database. To ensure accuracy and reliability, we specified the cleavage enzyme as Trypsin/P with allowance for up to two missing cleavages and up to five modifications per peptide. A mass error of 20 ppm was set for both precursor ions in the searches. To maintain high confidence and reliability, we set the false discovery rate (FDR) thresholds below 1% for proteins, peptides, and acetylated sites.

### 2.10 Bioinformatic analysis

We employed the ClustVis tool^3^ to conduct a principal components analysis (PCA) and characterize the CIH and CON groups. For differential expression analysis of acetylated proteins (DAPs), we applied strict criteria, including a *p*-value less than 0.05 and a fold change greater than 1.5 or less than 0.67, to identify DAPs. To analyze correlations of posttranslational modifications (PTMs) in our data, identify novel acetylation sites, and explore overlaps with other PTM types, we utilized the protein lysine modifications database (PLMD; version 3.0^4^) (Xu et al., 2017). Protein domains were analyzed using the InterPro database,^5^ while subcellular distribution prediction was performed using the Wolf Psort tool (version 1.0^6^). For functional enrichment analysis, we utilized the UniProt-GOA and Kyoto Encyclopedia of Genes and Genomes (KEGG) databases.^7^^,^^8^ To construct a protein-protein interaction (PPI) network, we used the STRING database (version 11.0^9^), and visualized the network in R using networkD3 (R package version 0.4). We selected interactions with a confidence score greater than 0.7 for DAPs. The MCODE plugin in Cytoscape software (version 3.6.1^10^) was utilized to identify the top five clusters with the highest degree of interconnectivity. We also examined the connections between DAPs and markers of gliosis (GFAP) and neurogenesis (DCX). To categorize acetylated sites based on their response to CIH, we employed a Gaussian mixture model. Furthermore, motif analysis of acetylated sites, considering the potential influence of neighboring conserved sequences on enzyme-substrate preference, was performed using the iceLogo tool (version 1.3.8^11^) (Colaert et al., 2009; Shen et al., 2022). We identified significant motifs within ± 6 amino acids surrounding lysine acetylation sites, using Mus musculus protein sequences as a reference. Additionally, prediction of secondary structure and surface accessibility was carried out using the NetSurfP tool (version 3.0^12^). The significance of both bioinformatic analyses was assessed using Fisher’s exact test with a corrected *p*-value < 0.05.

### 2.11 Immunoprecipitation and western blotting

For immunoprecipitation assays, the proteins extracted from mouse hippocampus were lysed using immunoprecipitation buffer (NP-40, Beyotime Biotechnology). Then, the samples were incubated with anti-acetyllysine antibody conjugated agarose beads (PTM Biolab) at 4°C overnight. After three washes with immunoprecipitation buffer, the acetylated proteins were centrifugated to pelletize beads (4°C, 60 s). The bound acetylated proteins were eluted by boiling in SDS loading buffer for 5 min. Samples were collected after centrifugation (4°C, 10,000 *g*). For Western blot assays, the immunoprecipitated proteins or input were separated on 12% SDS-PAGE gel. And then, the samples were transferred onto polyvinylidene fluoride (PVDF) membrane (Millipore, United States). The membranes were blocked for 1 h with 5% milk in TBST, and then incubated with primary antibodies: VDAC rabbit monoclonal antibody (diluted to 1:1000, Cell Signal Technology), 14-3-3 protein zeta/delta (Ywhaz) rabbit monoclonal antibody (diluted to 1:1000, Cell Signal Technology), and Camk2a rabbit monoclonal antibody (diluted to 1:1000, Cell Signal Technology) at 4°C overnight. Following three washes with TBST, the membranes were further incubated with horseradish peroxidase-conjugated secondary antibodies [Horse anti-mouse (diluted to 1:1000) and Goat anti-rabbit (diluted to 1:1000), ZSGB-Bio, Beijing, China] for 90 min at room temperature. Finally, the membranes were visualized using ECL (Beyotime Biotechnology, China).

### 2.12 Immunohistochemistry

Brain tissue was cut into 5-μm sections, and then were performed immunohistochemistry with anti-acetyl-Histone H3 (Lys27) antibody (H3K27ac), and anti-acetyl-Histone H3 (Lys9) antibody (H3K9ac). The sections were incubated overnight with primary antibodies against H3K27ac (diluted to 1:100, PTM Biolab), and H3K9ac (diluted to 1:50, PTM Biolab) at 4°C. Subsequently, the sections were incubated with a biotin-conjugated secondary antibody (diluted to 1:600, Thermo Fisher), followed by staining using a diaminobenzidine solution. The stained tissue sections in the hippocampal dentate gyrus (DG) region were examined by a light microscope. The average integral optical density (IOD) of selected fields were analyzed by Image J software.

### 2.13 Statistics

We used GraphPad Prism 8 software to compare the CIH and CON groups, considering a *p*-value of less than 0.05 as statistically significant. We presented the data in the format of mean ± SEM. For the NORT, Y-maze test, and MS/MS data, we performed an unpaired *t*-test to calculate statistical significance. In bioinformatics analysis, which included protein domain, GO, and KEGG pathway analysis, we employed a two-way Fisher’s exact test for calculation. To perform motif analysis, we utilized a binomial test, while we analyzed secondary structure distribution and surface accessibility through a Wilcoxon rank sum test.

## 3 Results

### 3.1 Hippocampal lesions and behavioral changes after chronic intermittent hypoxia

**FIGURE 1:**
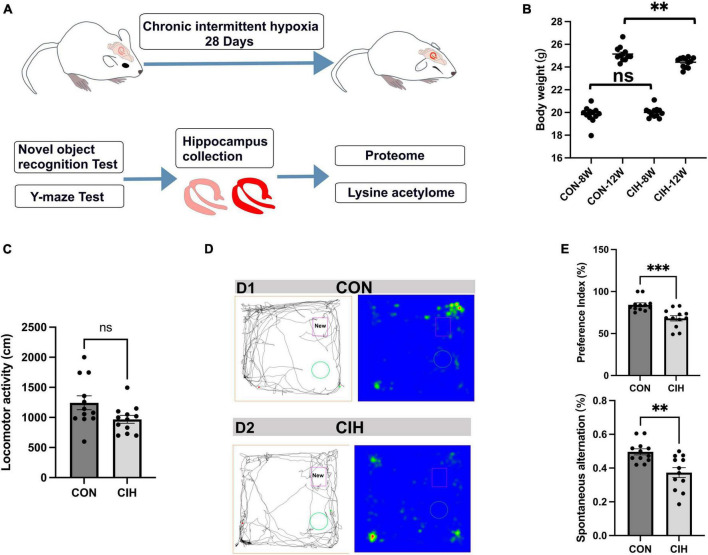
Decreased body weight, behavioral alterations, and hippocampal injury at 4 weeks post-CIH. **(A)** Experimental design. NORT, Novel object recognition test. **(B)** Statistical analysis for changes in body weights of the mice in CIH and CON groups upon 4 weeks of CIH intervention. Body weight was decreased during hypoxia (*n* = 12 mice/group). *p* = 0.0035 (unpaired *t*-test). **(C)** Bar charts shows locomotion after CIH or normoxia exposure. *n* = 12 for each group. Data are shown as mean ± SEM. ns: no significant. **(D,E)** Behavioral alterations were assessed by NORT and Y-Maze test (*n* = 12 mice/group). Typical movement tracks for CON (D1) and CIH (D2). Black lines indicate movement trajectories, whereas red hues denote the new object. Green circles indicate the old object. In the heatmap, areas with higher values of optical density indicate increased time spent by the mice in those regions. CIH significantly injured novel object recognition memory (*p* = 0.0005) and spatial working memory in the CIH group (*p* = 0.0017) when compared with the CON. Error bars represent mean ± SEM (*n* = 12 mice/group). ***p < 0.0001, **p < 0.005. ns: not significant; CON: Control; CIH: chronic intermittent hypoxia.

**FIGURE 2:**
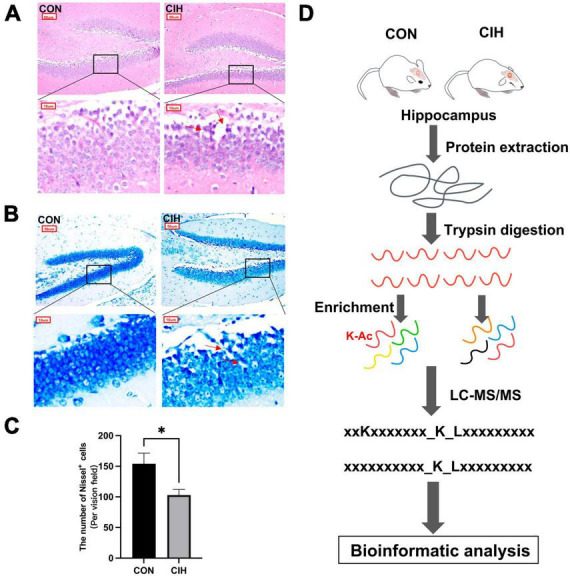
Pathological damage in CIH mice. HE staining **(A)** and Nissl staining **(B)** shows hippocampal morphological or structural abnormalities under CIH. The red arrow points to the damaged neuron body. Scale bar: 50 μm or 10 μm. **(C)** Quantification of the number of Nissl^+^ cells (*p* = 0.0262). Data are expressed as mean ± SEM (*n* = 6 mice/group). *p < 0.05 (unpaired *t*-test). **(D)** Bioinformatic analysis of proteome and acetylome. CON: Control; CIH: chronic intermittent hypoxia.

To characterize changes in cognition and lysine acetylation abundance specifically in the hippocampus in CIH mice, we treated male C57BL/6J mice with a gas control apparatus to simulate chronic intermittent hypoxia for 4 weeks (Figure 1A). Exposure to chronic intermittent hypoxia was found to influence the progression of body weight (Figure 1B; Supplementary Table 1). After 4 weeks of CIH exposure, we observed a substantial reduction in the body weight of the CIH group (24.42 g ± 0.44) compared to that of the control group (25.14 g ± 0.63). To further investigate the cause of weight loss, we also measured the physical activity and dietary intake. In the 16 h after the last day of daily exposure, the CIH group consumed an average of 13.92 ± 1.04 g of food, which was significantly less than that of the CON group (15.78 ± 0.38 g of food; *p* = 0.044), while both groups drank the same amount of water (CIH, 26 ± 1.73 ml; CON, 22 ± 3.46 ml; *p* = 0.148) (Supplementary Table 1). Locomotor activity did not significantly change in the CIH group compared to the normoxic CON group (Figure 1C). The above results indicate that the reduction in food intake is a significant factor contributing to the weight loss observed in the CIH group. The above results indicate that a reduction in food intake was a significant factor contributing to the weight loss observed in the CIH group. The NORT and Y-maze (Figures 1D, E) tests revealed that cognitive ability, such as learning or memory, was significantly impaired following CIH exposure (Supplementary Table 1). Learning and memory ability are intimately associated with the structure and morphology of hippocampal neurons. Consequently, we used hematoxylin and eosin (HE) staining and Nissl staining to reveal damage in the hippocampi of the mice. HE staining (Figure 2A) and Nissl staining (Figure 2B) revealed that exposure to chronic intermittent hypoxia resulted in cytolysis and cytoplasmic vacuolation in the hippocampal DG region compared to the control group. In the CON group, neurons exhibited round or oval cell bodies with clearly visible nuclei, while in the CIH group, hippocampal neurons were damaged and lost (Figure 2C).

### 3.2 CIH promotes the activation of glial cells, inhibits neurogenesis, and induces inflammation

**FIGURE 3:**
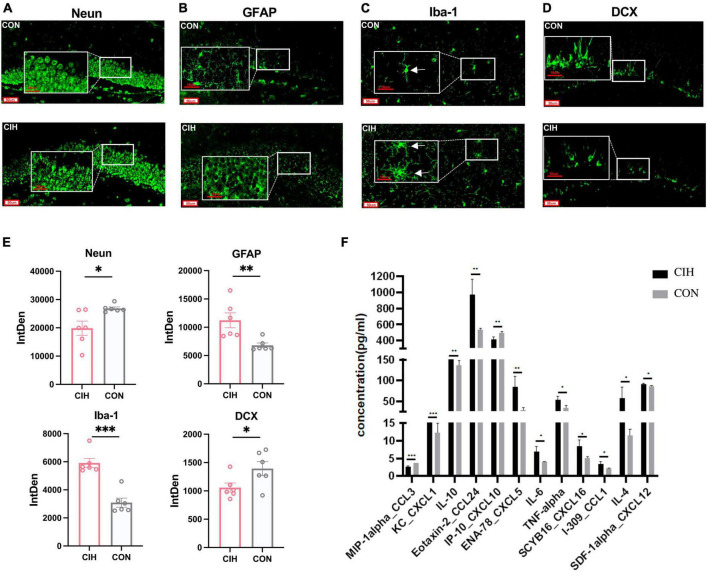
Representative immunofluorescence images. **(A–D)** Representative immunofluorescence images of Neun, GFAP, iba-1, and DCX for hippocampus when exposed to CIH. Scale bar: 50 μm or 10 μm. **(E)** Analysis of the immunofluorescence intensity of Neun, GFAP, iba-1, and DCX by measuring integrated density (IntDen) values. Data are expressed as mean ± SEM (*n* = 6 mice/group). **(F)** Cytokine and chemokine levels in the hippocampus of mice in the CON and CIH groups (*n* = 3 mice/group). Data are presented as means ± SEM. Statistical analysis was performed using student *t*-test, *p < 0.05, **p < 0.01, ***p < 0.001. CON: Control; CIH: chronic intermittent hypoxia.

NeuN is a marker protein for neuronal cells, and Iba-1 and GFAP are marker proteins of microglia and astrocytes, respectively. In our study, we performed immunofluorescence staining in the DG region of the hippocampus using NeuN, GFAP, Iba-1 and DCX antibodies, as shown in Figures 3A–D. Compared with those in the CON group, the staining intensity level of GFAP and Iba-1 in the CIH group were increased, which indicated potential glial activation (Figures 3B, C, E). Conversely, NeuN was notably decreased in the CIH group, as demonstrated in Figures 3A, E. The downregulated NeuN suggested a potential detrimental effect of CIH on neuronal integrity, as a reduced level of NeuN is generally associated with neuronal loss or dysfunction. To explore the effects of chronic intermittent hypoxic conditioning on neurogenesis, we performed DCX immunofluorescence staining. Compared with that in the CON group, the number of DCX-positive cells in the hippocampus was significantly lower in the CIH group (Figures 3D, E), suggesting that CIH inhibits neurogenesis.

Microglia are innate immune cells that participate in immune surveillance within the CNS. Astrocytes also play an active role in the regulation of neuroinflammation. To assess the overall inflammatory status of the hippocampus, we next investigated the cytokine and chemokine levels in the hippocampi of mice in the CON and CIH groups via a Luminex assay. The levels of many cytokines and chemokines, including CXCL5, CXCL16, IL-4, IL-6, IL-10, and TNF-α, were increased in the CIH group compared with those in the CON group (Figure 3F). These results indicated that CIH induced neuroinflammation. In summary, above results underscore the potential detrimental effects of CIH on hippocampus function.

### 3.3 Changes in the hippocampal proteome

Despite the observed cognitive decline and neural damage resulting from CIH exposure, the specific mechanisms underlying the perturbation of the hippocampal proteome and acetylome remain elusive. To address this gap, we employed MS methods to examine the alterations in protein abundance and acetylation in mice subjected to CIH (Figure 2D). To identify changes in the protein abundance and biological processes (BP) associated with the CIH response, we carried out proteomic analysis (Figure 2D; Supplementary Table 2) using a CIH model. As shown by the PCA, our data clearly distinguished the CIH group from the CON group (Supplementary Figure 1A). We identified 5,729 proteins in the hippocampi of CIH and CON mice, 4,878 of which were quantified via proteomic spectrogram analysis (Supplementary Figure 1B). Although numerous proteins were identified, only 21 proteins exhibited significant differences (Supplementary Figure 1C). Specifically, as shown in Supplementary Figure 1D, Clic6 and Orai2 were significantly regulated in the CIH group and were tightly associated with cognitive changes (Xu et al., 2020; Ma et al., 2021). We specifically focused on the abundance of lysine acetyltransferases (KATs) and lysine deacetylases (KDACs) via proteomics analysis. The proteomic data encompassed several KDACs (Sirt2-3, Sirt5, Hdac1-2, Hdac4-6, Hdac11) and KATs (Acat1-2, Atat1, Chat, Crat, Crebbp, Dlat, Naa10, Naa15, Naa25, Naa30, Naa35, Naa50, Nat10, Nat14). Although there were more types of KATs and KDACs, no statistically significant differences in protein abundance were observed (Supplementary Table 4). In BP analysis, the majority of proteins were enriched in phagocytosis and immune response, especially in B cells (Supplementary Figure 2A; Supplementary Table 3). Furthermore, in the analysis of cellular components (CC), most of proteins showed enrichment in immunoglobulin complexes (Supplementary Figure 2B; Supplementary Table 3). Finally, in the analysis of molecular functions (MFs), a notable enrichment of these proteins in immunoglobulin receptor-binding activities was observed (Supplementary Figure 2C; Supplementary Table 3).

### 3.4 Identification of lysine acetylation proteins and sites in the hippocampus

**FIGURE 4:**
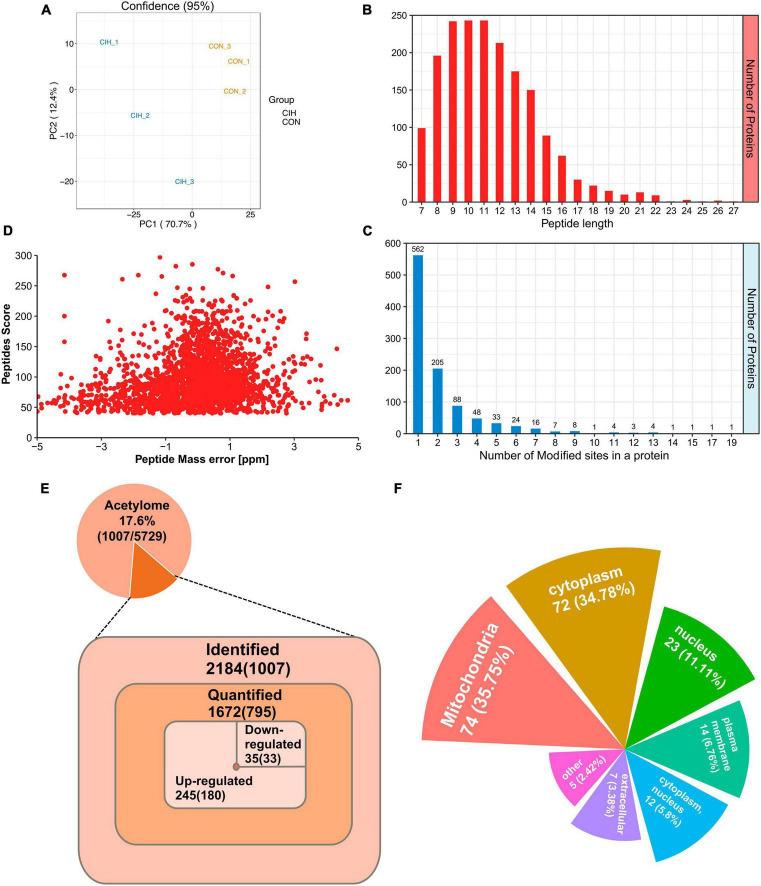
Profiling lysine acetylation proteome in the hippocampus. **(A)** Principal component analysis (PCA) of acetylome data generated from CIH and CON groups. **(B)** Distribution of peptide length of all identified acetylated peptides. **(C)** The number of acetylation sites within each modified protein. **(D)** Mass error distribution of all identified acetylated peptides. **(E)** The global view of the acetylated proteins and acetylation sites identified in the study (Top). The Venn diagram showed the number of acetylation sites and the corresponding proteins in brackets (Bottom). Significantly upregulated or downregulated proteins were defined as having a fold change > 1.5 or < 0.667 and *p* < 0.05. **(F)** Rose plots represent the cellular localization of significantly acetylated proteins after chronic intermittent hypoxia.

Despite the limited number of differentially abundant proteins identified in the hippocampus, we observed substantial increases in the abundance of acetylated proteins and sites. Similarly, we evaluated the quality of MS data first. To ensure the reliability and relevance of our analysis, we applied strict filtering criteria to the data. Specifically, we used the significance criterion of *p* < 0.05 and a fold change threshold of at least 1.5 to narrow down the list of potentially relevant changes in lysine acetylation levels. We performed PCA to characterize the signatures of and distinguish the CIH and CON groups (Figure 4A). According to our identification, the lengths of all the acetylated peptides ranged from 7 to 27 amino acids, and most of them ranged from 7 to 16 amino acids (Figure 4B). Additionally, the number of lysine acetylation sites in each protein ranged from 1 to 19 and 55.8% of acetylated proteins had only one lysine acetylation site (Figure 4C). Mass errors in the lysine acetylome were highly accurate (Figure 4D; Supplementary Table 5). In total, we identified 2,184 acetylation sites distributed across 1,007 acetylated proteins. These findings highlighted that approximately 17.6% of all the modified proteins exhibited acetylations (Figure 4E, Top). Among the 1,672 acetylated sites quantified on 795 proteins, the upregulated DAPs and differential acetylated sites (DASs) were the majority (Figure 4E, Bottom). Interestingly, Qian et al. (2022) observed an increase in cognitive function in mice and found that the downregulated acetylation sites were predominant. It seems that global changes in the hippocampal lysine acetylome could contribute to alterations in cognitive ability. Despite advances in the understanding of cognitive dysfunction, research exploring the impact of lysine acetylation on hippocampal models of cognitive changes is lacking. Additionally, those associated with mitochondria, such as Sptan1 (19 sites), Aco2 (18 sites), Cnp (15 sites), Idh2 (14 sites), and Got2 (13 sites), exhibited the most abundant acetylations. Furthermore, we predicted the subcellular distribution of DAPs upon CIH intervention; these proteins were localized mainly to mitochondria (35.75%), the cytoplasm (34.78%), and the nucleus (11.11%) (Figure 4F; Supplementary Table 6). Overall, these findings highlight the importance of acetylation in mitochondrial regulation and suggest their potential involvement in the cellular responses to CIH-induced damage. In addition, to further validate the reliability of our MS data, we performed Western blot analysis in this study (Supplementary Figures 3A, B). Western blot analysis conducted in our study also revealed that the levels of acetylated proteins (VDAC, Ywhaz, Camk2a) under CIH conditions were consistent with the results obtained from the MS data.

### 3.5 Posttranslational modification correlation analysis for acetylation sites

Determining novel lysine modification sites is crucial for expanding the understanding of PTMs, revealing new functionalities and regulatory mechanisms, and identifying potential therapeutic targets. By comparison with previously reported lysine modification sites in mice from the PLMD database, we identified 292 novel proteins and 977 newly discovered lysine acetylation sites (Supplementary Table 7). Furthermore, our analysis revealed the presence of various other types of PTMs at the identified lysine sites, including ubiquitination (920), succinylation (776), malonylation (604), and glutarylation (209) (Supplementary Table 8). Among them, the Ywhaz can undergo both acetylation and ubiquitination at sites K11, K120, and K138. Similarly, the Calcium/calmodulin-dependent protein kinase type II subunit delta (Camk2a) can be acetylated and ubiquitinated at sites K56, K136, K250, K258, K291, and K42 (Supplementary Table 7). Another example is fructose-bisphosphate aldolase (Aldoa), for which we newly discovered acetylation at the K208 site. These findings highlight the complexity of protein posttranslational modifications. Different types of modifications occurring at the same or adjacent sites on a protein can potentially result in crosstalk, which could contribute to the intricate nature of diseases.

### 3.6 CIH altered the characteristics of acetylation sites

**FIGURE 5:**
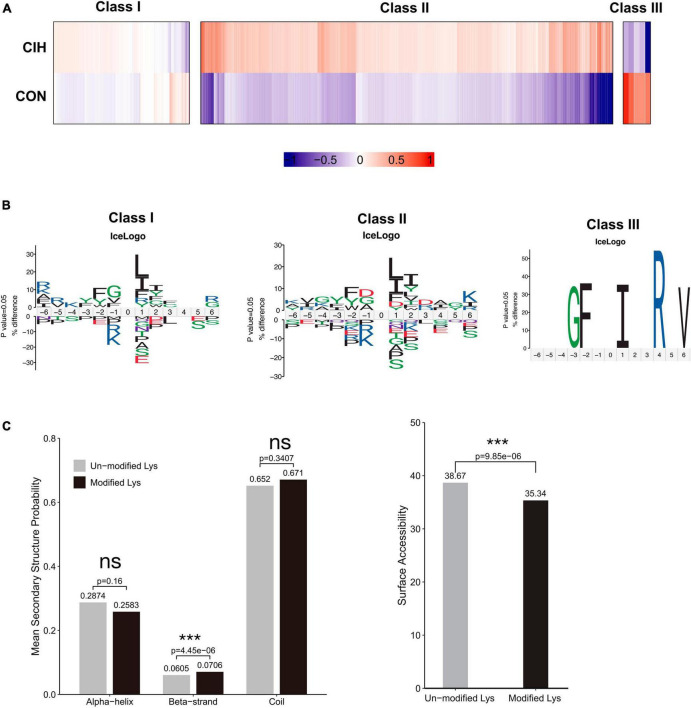
Motif analysis of all the identified sites. **(A)** Based on acetylated sites’ overall trends between the CIH and normoxia conditions, we grouped 3 classes. Class I sites exhibited no significant change. Class II were upregulated, and Class III sites were downregulated. **(B)** Predicted amino acid motifs for each group identified using a binomial test (*p* < 0.05). The motif showed significant amino acids surrounding each site flanking position 0 lysine. **(C)** Conformational tendencies of all differentially acetylated sites were predicted by protein secondary structures (left) and surface accessibility (right). After chronic intermittent hypoxia, the differentially acetylated sites were significantly enriched in Beta-strand (*p* = 4.45 × 10^–6^) and showed a significant decrease in surface-exposed accessibility (*p* = 9.85 × 10^–6^) by the Wilcoxon Rank Sum test. ***p < 0.001. ns: not significant; CON: Control; CIH: chronic intermittent hypoxia.

To examine the patterns of identified acetylated sites following CIH treatment, we divided all acetylated sites into 3 categories: class I contained unregulated sites, class II mostly contained sites upregulated by CIH, and class III mostly contained downregulated sites (Figure 5A). We conducted motif analysis on each group to gain a better understanding of their physical properties (Figure 5B). We found that class II sites favored aspartic acid (D) at the −1 position, while class I sites favored glycine (G) at the −1 position. Furthermore, we observed that class III sites preferred isoleucine (I) at the +1 position, while leucine (L) was favored at both class I and II sites. Compared to non-modified lysine residues, DASs were significantly enriched in beta strands (*p* = 4.45 × 10^–6^; Figure 5C, left). Similarly, DASs exhibited greater accessibility to surface exposure (Figure 5C, right). These results suggest that DASs caused by chronic intermittent hypoxia in the hippocampus may impact protein function by changing preferences for neighboring amino acids, beta-strand secondary structure and surface accessibility.

### 3.7 Profiling the lysine acetylome in mouse models of CIH

**FIGURE 6:**
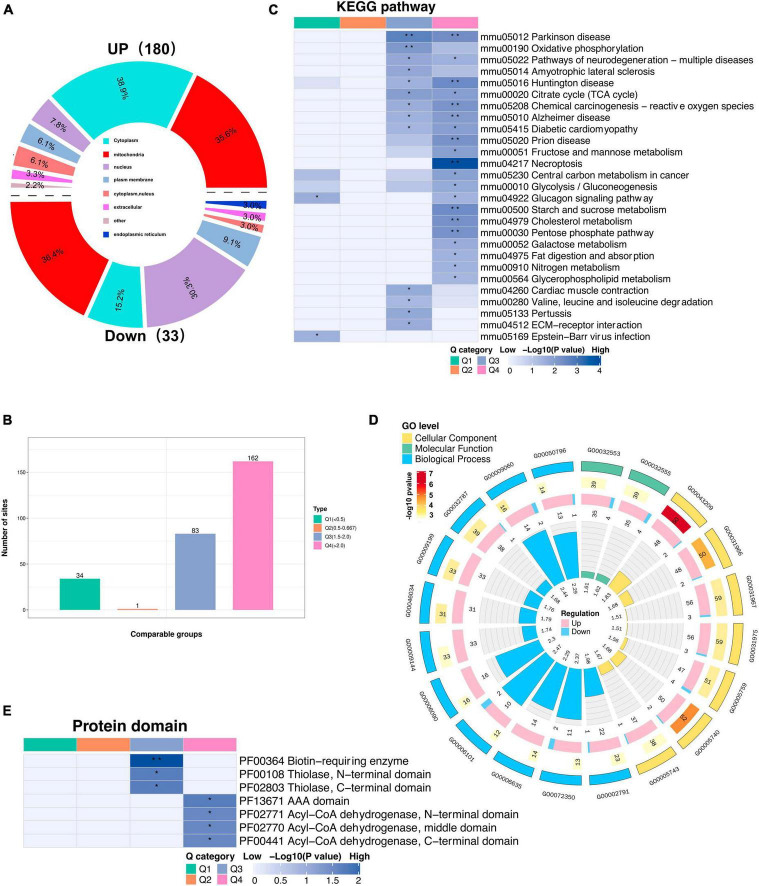
Subcellular location and KEGG/GO analysis of differentially expressed acetylated proteins. **(A)** Rose diagram showed the number of all different acetylated proteins in different subcellular structure species, and the corresponding subcellular structure information is shown in different colors. **(B)** The acetylated proteins were divided into four groups based on the fold-change values, respectively, named Q1 to Q4, and enriched with KEGG pathway and protein domain analysis, respectively. **(C)** The bar plot showed the major enriched KEGG pathways by acetylated proteins. **(D)** The Circular plot representing the GO enrichment of differentially acetylated proteins. Each circle means (from the outside to the inside): the First Circle: is the enriched GO function, with different colors representing different categories; the second circle: is the function that contains different acetylated protein numbers and significance *p*-value values, color represents the enrichment significance *p*-value after-Log10 transformation; the third circle: up and down-regulated differential acetylated protein number bar graph; the fourth circle: fold enrichment of each function after Log2 transformation. **(E)** The bar plot shows the major protein domains enriched by the acetylated proteins. *p < 0.05, **p < 0.01.

To gain insight into the biological functions and networks associated with differentially acetylated lysine residues, we performed domain, GO/KEGG pathway, and subcellular localization analyses. Interestingly, we observed a significant increase in the number of upregulated DAPs localized to mitochondria compared to the number of downregulated DAPs when performing subcellular localization analysis separately for upregulated and downregulated proteins (Figure 6A, Supplementary Table 6). These findings further implies potential dysregulation of mitochondrial function in response to CIH. Such dysregulation may contribute to cognitive impairments. Next, we categorized the significantly changed acetylated sites into four groups (Q1-Q4) based on the degree of fold-change values observed (Figure 6B). For the KEGG pathways, DAPs in Q3 and Q4 were mainly enriched in TCA cycle, necroptosis, and neurodegeneration diseases (Parkinson’s disease, Alzheimer’s disease, and Huntington disease) (Figure 6C). For domain enrichment analysis (Figure 6E), DAPs in Q4 were mainly enriched in Acy-CoA dehydrogenase, while in Q3 they were mainly enriched in Biotin-requiring enzyme. The Acy-CoA dehydrogenase domain and the main KEGG pathways mentioned above are closely associated with mitochondria. Consistent with these above findings, GO enrichment analysis showed that the main biological process, molecular function, and cellular component were closely associated with mitochondrial activity. This includes GO:0032787 (monocarboxylic acid metabolic process), GO:0006090 (pyruvate metabolic process), and GO:0005759 (mitochondrial matrix) (Figure 6D). Both the subcellular localization analysis and functional pathway analyses consistently indicate that mitochondrial function is disrupted by CIH. The altered acetylation of proteins associated with mitochondrial function are likely significant contributors to the cognitive impairments observed in response to CIH.

### 3.8 Analysis of the functional enrichment of differentially acetylated mitochondrial proteins

**FIGURE 7:**
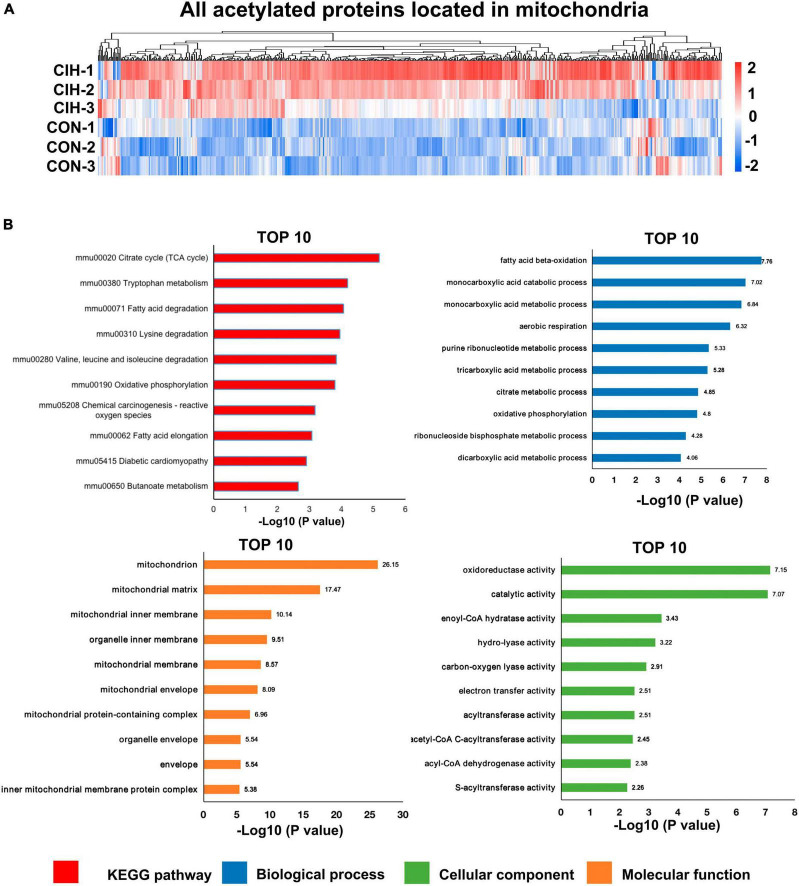
Functional pathway enrichment analysis of differentially regulated mitochondrial-localized proteins. **(A)** Heat map displaying differential acetylated proteins localized to mitochondria. The hippocampal samples were represented in rows, and the protein was delineated in columns. The color bar at the bottom of the figure showed that red indicated up-regulation and blue indicated down-regulation. **(B)** Bar plots are presented showing the KEGG pathways, as well as GO analysis results revealing biological processes, cellular components, and molecular functions of the differentially acetylated proteins localized to mitochondria.

Moreover, accumulating evidence has also demonstrated that mitochondrial dysfunction plays a pivotal role in the pathophysiology of cognitive impairment (Imai and Guarente, 2014; Bonkowski and Sinclair, 2016; Sorrentino et al., 2017; Song et al., 2021). Consistent with above evidence, we found that most acetylated sites were in mitochondrial proteins and were highly upregulated after chronic intermittent hypoxia (Figure 7A). Among the identified KEGG pathways (Figure 7B), the citrate cycle (TCA cycle) was the most highly enriched. The TCA cycle serves as a central hub in cellular metabolism due to its ability to accept multiple substrates. The metabolites of the TCA cycle are essential for protein synthesis. Furthermore, it is increasingly recognized that the metabolites of the TCA cycle also participate in regulating DNA methylation, histone modifications, and PTMs of proteins to modulate their function (Martínez-Reyes and Chandel, 2020). Taken together, the GO analysis of the DAPs revealed that these proteins are involved mainly in monocarboxylic acid catabolic processes, fatty acid beta-oxidation, and oxidoreductase activity, among other pathways (Figure 7B), consistent with the KEGG annotation results.

### 3.9 Analysis of protein-protein interaction networks

**FIGURE 8:**
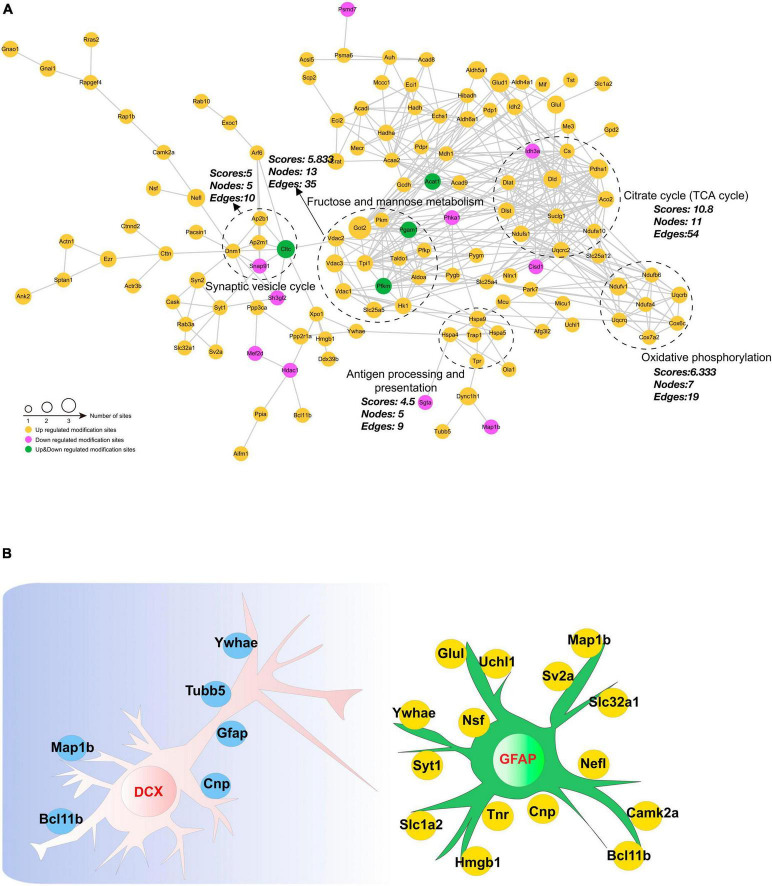
Interaction network encompassing the differentially acetylated proteins. **(A)** Network diagram of differentially acetylated proteins and their interactions. The five most highly connected subnetworks are circled for visualization. **(B)** Network methods relying on known protein-protein interactions identify proteins relating to GFAP and DCX (markers of adult gliosis and neurogenesis).

Investigating protein-protein interactions (PPI) is a crucial step in uncovering the functions of proteins, enabling the study and manipulation of pivotal cellular processes. In order to gain a better understanding of the interactions among DAPs, we conducted a PPI network analysis to display the interconnections among 213 DAPs with high confidence (Figure 8A). In this analysis, highly clustered proteins often share similar or related functions. We identified the five most highly connected clusters: the TCA cycle, oxidative phosphorylation, fructose and mannose metabolism, the synaptic vesicle cycle, and antigen processing and presentation. In the TCA cycle cluster, Aco2, Ndufa10, Pdha1, and Ndufs1 were highly acetylated while their protein contents remained unchanged. In the fructose and mannose metabolism, we observed Voltage-dependent anion channel (Vdac)1–3 were highly acetylated. These proteins were documented to exhibit a strong correlation with cognitive dysfunction (Akarsu et al., 2014; Mangialasche et al., 2015; Huang et al., 2021). Neurogenesis-glia interactions play an important role in hippocampal function (Kim et al., 2020). Based on our findings that CIH suppresses neurogenesis and enhances astrocyte activation, we further investigated the potential relationships between DAPs and GFAP or DCX (Figure 8B). The results showed that several DAPs were associated with DCX or GFAP, indicating that acetylation may have a significant impact on neurodevelopmental disorders during CIH.

### 3.10 Sodium butyrate regulates acetylation to ameliorate cognitive impairment

To enhance a connection between acetylation and CIH-induced hippocampal damage, we employed a histone deacetylase inhibitor, NaB, for intervention. NaB has recently been recognized as a potential neuroprotective agent for various neurodegenerative diseases (Sharma et al., 2015). After intraperitoneal injection of NaB (Supplementary Figure 3C), we observed an improvement in cognitive function in the CIH+NaB group compared to the CIH group, as assessed by the NORT (Supplementary Figure 3D). We hypothesized that histone acetylation may be linked to CIH hippocampus injury. We tested this hypothesis by examining the acetylation levels of H3K9 and H3K27 in hippocampus from the three groups. The results revealed that H3K9 and H3K27 acetylation were suppressed in the CIH group, while treatment with NaB rescued its acetylation (Supplementary Figure 3E). These findings further confirm the role of acetylation in the cognitive impairment caused by CIH.

## 4 Discussion

Chronic intermittent hypoxia, a prominent characteristic of OSA, has been shown to induce cognitive decline in mice. However, the precise mechanisms responsible for this phenomenon are yet to be fully investigated. In this study, we investigated the changes in proteome and acetylome profiles in a CIH model. Proteome analysis revealed that only a few proteins, including Clic6 and Orai2, exhibited alterations (Supplementary Figure 1D), which were associated with cognition. However, no acetylations were identified on these two proteins, leading us to not delve into their discussion. Furthermore, our observations suggest that CIH resulted in a relatively limited number of global protein changes. In contrast, DAPs exhibited more significant and widespread alterations (Figure 4E). In recent years, there has been increasing research interest in the influence of PTMs, particularly acetylation, on cognitive function (Jaenisch and Bird, 2003; Perikleous et al., 2018). However, few studies have directly shown that PTM changes are responsible for long-lasting behavioral effects in CIH. Therefore, this study primarily focused on investigating and discussing the impact of acetylation on cognitive impairment induced by CIH (Figures 1A, 2D).

We identified 2,184 lysine acetylation sites on 1,007 proteins (Figure 4E). We further analyzed the quantified sites and proteins to identify those that were differentially regulated by CIH exposure. Our findings revealed that 280 of the identified acetylation sites located on 213 unique proteins were differentially regulated by CIH exposure. These results suggest that CIH exposure has significant effects on the acetylome profile of the hippocampus and may play a role in the pathogenesis of related cognitive impairments. We then conducted KEGG pathway analysis to gain insight into the potential molecular mechanisms underlying cognitive impairment induced by chronic intermittent hypoxia (CIH) exposure. Our analysis revealed that the pathways associated with the genes exhibiting the most enrichment were related to Parkinson’s disease, necroptosis, and oxidative phosphorylation (Figure 6C). Further investigation of these pathways revealed significant alterations in neurodegeneration-related diseases such as Alzheimer’s disease, Parkinson’s disease, and Huntington’s disease, as well as central carbon metabolism pathways including the TCA cycle, glycolysis/gluconeogenesis, and the pentose phosphate pathway. Additionally, we analyzed the protein domains enriched in the DAPs identified in our study, which revealed that the DAPs were primarily enriched in biotin-requiring enzymes and Acy-CoA dehydrogenase domains, further highlighting the potential role of lysine acetylation in regulating energy metabolism and cellular processes associated with cognitive function (Figure 6E). These findings provide novel insights into the complex molecular mechanisms underlying CIH-induced cognitive impairment and may have significant implications for the development of targeted interventions for related neurological disorders. However, further research is needed to validate these findings and identify specific protein targets for therapeutic intervention.

In addition to being involved in metabolic pathways, the necroptosis pathway was also significantly enriched in DAPs (Figure 6C), which are related to markers of neurogenesis (Figure 8B). Studies have suggested that necroptosis can be activated in response to ischemic brain injury, neuroinflammation, or neurodegenerative disorders such as Alzheimer’s disease. In the hippocampi of CIH mice, we observed increases in the expression levels of inflammatory factors, including IL-4, IL-10, and TNF-a (Figure 3F and Supplementary Table 9). The involvement of the necroptosis pathway in the hippocampus might contribute to neuronal loss or dysfunction through neuroinflammation. Additionally, we observed acetylation of K250 in Camk2a protein within the necroptosis pathway. Camk2a plays crucial roles in synaptic plasticity, learning, and memory processes within the hippocampus. PTMs of the Camk2a protein have been implicated in various aspects of hippocampal function (Küry et al., 2017). Phosphorylation of the T286 residue in Camk2a has been demonstrated to be crucial for neuronal function and development (Küry et al., 2017). Camk2 activation is not only necessary but also sufficient for the induction of long-term potentiation (LTP) in the hippocampus. LTP is an index of hippocampal functional plasticity (Poggini et al., 2023). Additionally, the histidine residue at position 282 in Camk2a has been identified as an important inhibitory amino acid residue that effectively suppresses Camk2a activity (Smith et al., 1992). PTMs at specific sites in Camk2a are known to play crucial roles in neuronal plasticity. However, further investigations are needed to determine the specific impact of K250 acetylation on hippocampal synaptic plasticity in Camk2a protein. Additionally, it remains to be determined whether there is any cross-talk or interplay between these neighboring sites and whether different modifications occur at the same sites.

Increasing evidence has indicated that CIH damages cognitive function through mitochondrial dysfunction in the brain (Laouafa et al., 2019). Both subcellular localization and functional pathway analyses have consistently indicated that mitochondrial function is disrupted by CIH. Mitochondria are the main organelles in cells that consume oxygen for energy production and metabolism; therefore, oxidative phosphorylation is affected by a lack of oxygen (Scharping et al., 2021). Lysine acetylation is a common posttranslational modification observed in enzymes associated with intermediate metabolism. In our study, 35.75% of the DAPs were found in mitochondria (Figure 4F), and most of these DAPs were upregulated (Figure 6A). Moreover, significant enrichment of both the TCA cycle and oxidative phosphorylation pathways was observed (Figure 7B). Oxidative phosphorylation is the process by which ATP is generated through electron transfer via the electron transport chain, which includes complexes I, III, and IV (Vercellino and Sazanov, 2022). Interestingly, the acetylation levels of key proteins belonging to the electron transport chain, such as Cox6c, Cox7a2, and Ndufa4, were increased in our study (Figure 8A). In addition, several subunits of ATP synthase (Atp5pd, Atp5po, and Atp5f1a) were also increased in our study. Moreover, our study revealed that CIH induces the upregulation of multiple acetylation sites on Vdac1, which is involved in fructose and mannose metabolism (Figure 8A; Supplementary Figure 3B). Vdac1, the most abundant protein on the outer mitochondrial membrane, is a vital protein that regulates mitochondrial function. Increased levels of Vdac1 have been shown to be associated with the progression of diseases involving cognitive impairment, such as Alzheimer’s disease and neonatal hypoxia–ischemia (Shoshan-Barmatz et al., 2010; Xue et al., 2021). Vdac1 undergoes PTMs due to oxidative stress, which is another critical pathological factor in Alzheimer’s disease development. Oxidative damage in the brains of neurodegenerative patients caused by nitration and carbonylation of Vdac1 may impair channel function, promote the pathogenesis and progression of brain disease, and contribute to cognitive impairment (Shoshan-Barmatz et al., 2010). Changes in the phosphorylation state of Vdac have also been observed in neurons of patients with cognitive impairment; these changes disrupt glucose metabolism, promote mitochondrial dysfunction, and activate cell apoptosis (Verma et al., 2022). Dysregulation of Vdac1 and its PTMs are implicated in impaired energy metabolism, oxidative stress, and neurodegenerative processes associated with cognitive impairment. Understanding the role of these modifications in Vdac1 could offer insights into therapeutic strategies targeting mitochondrial function and oxidative stress in the neurogenesis process. Furthermore, our study revealed that, when exposed to CIH, Got2, a crucial enzyme involved in mitochondrial metabolism, undergoes acetylation at six lysine residues, K90, K82, K302, K309, K396, and K404. Our findings align with existing evidence that acetylation inhibits the activity of mitochondrial enzymes (Qian et al., 2022). Moreover, our findings suggest that chronic intermittent hypoxia (CIH)-induced lysine acetylation may have detrimental effects on the mitochondrial tricarboxylic acid (TCA) cycle and oxidative phosphorylation pathways within the hippocampus. This disruption may lead to inefficient energy use, supporting the notion that CIH has damaging effects on cognitive function. By altering the activity of enzymes involved in these metabolic pathways, CIH-induced acetylation may decrease the efficiency of ATP production and contribute to mitochondrial dysfunction. This dysregulation can exacerbate oxidative stress and impair cellular processes crucial for normal brain function. Therefore, our findings provide new insights into the potential mechanisms underlying CIH-induced cognitive impairment and further highlight the importance of the proper regulation and maintenance of mitochondrial function in maintaining cognitive health.

Both animal and human studies have indicated that cognitive decline and memory problems are associated with low glucose metabolism in the brain (Ding et al., 2013; Zhang et al., 2021). Although this process occurs differently in different parts of the brain, it is mainly associated with regions that affect learning, memory, and behaviour (Pawlosky et al., 2017). Like in the TCA cycle and during oxidative phosphorylation, every enzyme in glycolysis is acetylated (Zhao et al., 2010). In accordance with the findings of a previous study, we observed increases in the acetylation of K783 and K819 on hexokinase (Hk1) and K208 on Aldoa and in K14 and K149 on triosephosphate isomerase (Tpi1) following CIH exposure. The increased acetylation of these critical glycolytic enzymes may inhibit their activity (Pei et al., 2022), further reducing glucose availability for energy production and contributing to cognitive impairment. Moreover, inflammatory activation of glial cells often leads to a metabolic shift from oxidative phosphorylation to aerobic glycolysis (Cheng et al., 2021). This metabolic switch may exacerbate the effects of CIH-induced glycolytic enzyme acetylation, further impairing cognitive function. Therefore, proper regulation of cellular metabolism is important for maintaining optimal cognitive health.

Hypoxia can also diminish cell viability in both glial cells and neurons (Wang et al., 2014). Effective communication between neurons, astrocytes, and microglia is crucial for the brain’s functional organization (Lana et al., 2020). Dysregulation of energy metabolism in neurons and glial cells may contribute to the pathophysiology of neurodegeneration, particularly under conditions of hypoxia–ischemia. One study demonstrated that oligodendrocytes enhance axonal energy metabolism by delivering SIRT2 to deacetylate mitochondrial proteins (Chamberlain et al., 2021). Elevated HDAC2 modifies transcription in hippocampal neurons and impacts microglial activity during neuroinflammation-induced cognitive impairment (Sun et al., 2019). Our results showed that several DAPs, including Camk2a, Hgmb1, and Glul, were related to GFAP or DCX (Figure 8B). These findings suggested that acetylation may be crucial in neurodevelopmental disorders in individuals exposed to CIH and indicate that CIH has the potential to modulate various biological functions via alterations in the acetylation levels of pertinent proteins in neurons and glial cells, possibly helping the hippocampus adapt by reshaping its function.

Several recent studies have shown that memory can be modulated by manipulating histone modifications via the use of HDAC inhibitors during memory formation, consolidation, and reconsolidation (Vinarskaya et al., 2021). NaB is an HDAC inhibitor that affects various types of brain damage (Lee et al., 2019). Furthermore, NaB, which can cross the blood-brain barrier and affect the epigenetic machinery in the brain, has been shown to ameliorate reductions in novel object memory when administered intraperitoneally (Jung et al., 2016). In this study, we intraperitoneally administered NaB to CIH group mice. The results revealed an improvement in memory in the CIH group. Additionally, we observed significant fluctuations in the levels of H3K27ac and H3K9ac (Supplementary Figure 3E). These findings provide further evidence for the significant role of acetylation in the cognitive impairment associated with CIH.

The current study had certain limitations. First, the number of participants was limited due to budgetary and ethical reasons. Increasing the sample size would help decrease biological variability. Second, we used only male mice to establish the CIH model to perform the studies described here. However, to avoid potential sex bias, we will consider using both male and female mice in future studies. Additionally, studies focused on the mechanisms of key acetylated proteins are lacking. In the future, we plan to concentrate on functional studies.

In summary, our study aimed to uncover the molecular mechanisms responsible for cognitive dysfunction induced by CIH. To this end, we performed lysine acetylome profiling to create a comprehensive and detailed landscape of lysine acetylation in the hippocampus. We discovered 2,184 lysine acetylation sites across 1,007 proteins. CIH differentially regulated 280 acetylated sites on 213 proteins, 35.75% of which were acetylated in mitochondria. Our findings suggest that oxidative phosphorylation, the TCA cycle, and glycolysis, which are located primarily in mitochondria, may exacerbate cognitive impairment following CIH. Overall, investigating the mechanisms underlying CIH-induced changes in hippocampal acetylation can aid in the development of scientific prescriptions for cognitive decline caused by CIH.

## Data availability statement

The data presented in the study are deposited in the ProteomeXchange repository, accession numbers PXD049225 and PXD049226.

## Ethics statement

The animal study was approved by the Capital Institute of Pediatrics’ Ethics Committee on Animal Care and Use. The study was conducted in accordance with the local legislation and institutional requirements.

## Author contributions

FL: Data curation, Formal analysis, Investigation, Methodology, Writing—original draft. WY: Data curation, Methodology, Writing—original draft. CC: Data curation, Software, Writing—original draft. YZ: Methodology, Supervision, Writing—original draft. YK: Methodology, Software, Writing—original draft. XH: Data curation, Methodology, Software, Writing—original draft. PP: Formal analysis, Validation, Writing—original draft. SW: Funding acquisition, Project administration, Resources, Visualization, Writing—review and editing. TZ: Resources, Supervision, Visualization, Writing—review and editing.

## Footnotes

1
http://imagej.nih.gov/ij/

2
http://www.maxquant.org/

3
http://biit.cs.ut.ee/clustvis/

4
https://cplm.biocuckoo.cn/

5
https://www.ebi.ac.uk/interpro/

6
https://wolfpsort.hgc.jp/

7
http://www.ebi.ac.uk/GOA/

8
https://www.kegg.jp/

9
https://string-db.org/

10
https://www.cytoscape.org/

11
https://iomics.ugent.be/icelogoserver/

12
https://services.healthtech.dtu.dk/services/NetSurfP-3.0/
