# Supplementary material for: Acetylome analyses provide novel insights into the effects of chronic intermittent hypoxia on hippocampus-dependent cognitive impairment
Source: Front Mol Neurosci. 2024 Feb 22;17:1324458. doi: 10.3389/fnmol.2024.1324458 (PMC10917988; doi:10.3389/fnmol.2024.1324458)
Supplement: Supplementary file 1 [file Table_1.docx]

Supplementary Material

Analysis of the lysine acetylome in the Hippocampus of Chronic intermittent hypoxia mice

Fan Liu^1,2,3^, Weiheng Yan^1^, Chen Chen^2^, Yubing Zeng^1,2^, Yaru Kong^1^, Xuejia He^4^, Pei Pei^2^, Shan Wang^1,2,3,4*^, Ting Zhang^1,2,3,4*^

*** Correspondence:** Shan Wang: wsaquarius@sina.com; Ting Zhang: zhangtingcv@126.com.

**Supplementary Figure 1.** Profiling proteome in hippocampi. (A). Principal component analysis (PCA) of proteome data to distinguish the CIH group from the CON group. Three biological replicates (bilateral hippocampi from every two mice as one replicate) were performed in each group. (B). The basic statistical figure for MS results. (C). The bar plot displays the number of differentially abundant proteins between the CIH group and the CON group. (D). Abundance profiles of proteins from Orai2 and Clic6 (unpaired t-test). Orai2 expression was significantly higher in the CIH mice compared with the CON. The intensity of Clic6 showed an opposite trend. CON: Control; CIH: Chronic intermittent hypoxia. *p < 0.05, ***p <0.001.

**Supplementary Figure 2.** GO analysis of differentially abundant proteins. (A). The bar plot showed the major enriched biological processes (BP) by different expressed proteins. (B). The bar plot showed the major enriched cellular components (CC) analysis by different expressed proteins. (C). The bar plot showed the major enriched molecular functions (MF) analysis by different expressed proteins. The horizontal axis represents the -Log10 transformed enrichment significance P-value, where higher values indicate stronger enrichment significance.

**Supplementary Figure 3.** Confirmation of the altered K-ac proteins and NaB supplementation. (A-B). Immunoprecipitaion and Western-blot confirmations of the altered K-ac proteins including Ywhaz, VDAC, Camk2a in the hippocampi of CIH and CON group mice (n = 3 mice/group). (C). Illustration of NaB injections into the CIH group mice. (D). Behavioral alterations were assessed by NORT test (n = 12 mice/group). (E). Photomicrographs of the immunohistochemical staining of H3K27ac and H3K9ac in the hippocampus of mice were obtained, and the expression level of was analyzed by measuring the IOD/Area values (n = 5 mice/group). Scale bar: 50 μm. Data are expressed as mean ± SEM, ***p < 0.0001, **p < 0.005, *p < 0.05 (one-way ANOVA analysis). ns: not significant, CON: Control; CIH: chronic intermittent hypoxia; NaB:Sodium butyrate; IOD:integral optical density.
